# Editorial: The role of code-modulated evoked potentials in next-generation brain-computer interfacing

**DOI:** 10.3389/fnhum.2025.1548183

**Published:** 2025-02-20

**Authors:** Víctor Martínez-Cagigal, Jordy Thielen, Roberto Hornero, Peter Desain

**Affiliations:** ^1^Biomedical Engineering Group (GIB), E.T.S Ingenieros de Telecomunicación, University of Valladolid, Valladolid, Spain; ^2^Centro de Investigación Biomédica en Red en Bioingeniería, Biomateriales y Nanomedicina (CIBER-BBN), Madrid, Spain; ^3^Donders Institute for Brain, Cognition and Behaviour, Machine Learning & Neural Computing, Radboud University, Nijmegen, Netherlands

**Keywords:** brain-computer interfacing (BCI), event-related potentials (ERP), electroencephalogram (EEG), code-modulated visual evoked potential (c-VEP), machine learning, pseudo-random noise-codes, signal processing, closed loop

Research on brain-computer interfaces (BCIs) using the code-modulated evoked potential (c-VEP) has recently achieved remarkable advancements (Martínez-Cagigal et al., 2021). These breakthroughs are attributed to the sophisticated design of the stimulus protocols and the innovative decoding techniques, which together establish c-VEP-based BCIs as the current state-of-the-art for communication and control applications. This Research Topic aimed to propel the field forward by fostering original contributions, with a specific focus on improving the usability, reliability, and practicality of c-VEP-driven BCI systems. The goal was to bring greater attention to this emerging field, which, despite its notable achievements, still needs to foster broader adoption of these technologies in both clinical settings and everyday life.

The c-VEP stimulus protocol is distinctly different from other major classes of evoked responses, such as the event-related potential (ERP) and steady-state visual evoked potential (SSVEP) (Martínez-Cagigal et al., 2021). The ERP protocol, typically based on an oddball paradigm, operates at a much slower pace, with a typical stimulus onset asynchrony (SOA) of ~250 ms (4 Hz) compared to the significantly faster SOA of at least 16 ms (60 Hz) used in c-VEP. Similarly, while the SSVEP paradigm is also relatively fast as compared to ERP, SSVEP protocols rely on a frequency-tagging approach, where stimuli are restricted to periodic signals with specific frequencies and phases. In contrast, the c-VEP protocol employs a noise-tagging approach, allowing for a much wider range of stimulus sequences, including non-periodic patterns, while also demonstrating greater resilience to narrowband interferences. Moreover, recent evidence has revealed that, from an information-theoretic perspective, the maximum information transfer rate achievable via the visual-evoked pathway in c-VEP-based BCIs significantly exceeds that of SSVEP-based systems (Shi et al., 2024).

Furthermore, the c-VEP field places significant emphasis on designing stimulus sequences to ensure that the resulting neural responses are (near-)orthogonal, thereby facilitating and enhancing decoding performance. Initially, the field tested carefully selected pseudorandom binary codes from telecommunications, such as the well-established m-sequence and Gold codes (Martínez-Cagigal et al., 2021). At present, researchers are increasingly exploring alternative noise codes with advantageous properties, aiming to improve signal-to-noise ratio (SNR), enhance decoding accuracy, or increase user comfort (e.g., Martínez-Cagigal et al., 2023). However, achieving optimal structure of discernibility in the stimulus domain does not necessarily carry over to the response space, where the real-time command decoding ultimately takes place.

Complementing the rapid and optimized stimulus presentation, dedicated decoding approaches have been developed to capitalize on the rapid, repeated but pseudorandom presentation of flashes. These methods significantly reduce, and in some cases even eliminate, the need for subject-specific training data to calibrate classifiers (e.g., Thielen et al., 2021). Additionally, advances in decoding techniques, including dynamic stopping, asynchronous operation, and non-control state detection, enable quick, reliable, and intuitive selection of target classes during online BCI use. In this Research Topic, Ahmadi et al. introduced a novel Bayesian dynamic stopping method aimed at optimizing the trial duration during online use. One of the innovative features of their method is its ability to intuitively fine-tune and control its behavior based on the specific application requirements.

Moreover, earlier c-VEP studies primarily relied on binary noise codes that encoded stimuli through contrast reversals, typically at full contrast, alternating between black and white (Martínez-Cagigal et al., 2021). While these approaches achieved high performance, the community quickly recognized the trade-off with maintaining a high level of user comfort. Currently, research increasingly focuses not only on identifying optimal stimulus sequences, such as non-binary codes (e.g., Martínez-Cagigal et al., 2023) or those using white noise codes (e.g., Miao et al., 2024), but also on improving the visual characteristics of the stimuli, for example by employing burst codes and textured stimuli (Dehais et al., 2024). Optimizing both the stimulus sequences and their appearance contributes to enhanced BCI performance as well as an improved user experience. In this Research Topic, Fernández-Rodríguez et al. explored the effects of varying spatial frequencies in checkerboard-like stimuli on both performance and user experience in c-VEP-based BCIs. Their findings again stress the importance of customizing visual stimuli to optimize both system performance and user satisfaction.

Finally, research on c-VEP-based BCIs has often focused on the speller application for communication as a benchmark (Martínez-Cagigal et al., 2021). However, this emphasis overlooks the vast potential of c-VEP for a wide range of applications beyond the standard controlled lab environment. Many promising uses of c-VEP-based BCIs exist that could offer significant societal benefits. In this Research Topic, we highlight two such innovative applications. First, Huang et al. explored the potential of c-VEP for biometric authentication by integrating it into a mild-burdened cognitive task. Second, Moreno-Calderón et al. introduced a multiplayer competitive video game that uses a c-VEP-BCI to implement the classic game of “Connect Four."

In conclusion, the four studies featured in this Research Topic mark significant advancements in addressing the challenges associated with c-VEP-based BCIs. By optimizing stimulus protocols, refining decoding techniques, and demonstrating practical real-world applications, these contributions lay the foundation for a new generation of BCIs that are more reliable, faster, user-friendly, and accessible to a broader range of users and use cases. Continued interdisciplinary collaboration will be crucial in transforming these innovations into impactful, plug-and-play solutions in the near future.
